# Normalized Polarization Ratios for the Analysis of Cell Polarity

**DOI:** 10.1371/journal.pone.0099885

**Published:** 2014-06-25

**Authors:** Raz Shimoni, Kim Pham, Mohammed Yassin, Mandy J. Ludford-Menting, Min Gu, Sarah M. Russell

**Affiliations:** 1 Centre for Micro-Photonics, Faculty of Science, Engineering and Technology, Swinburne University of Technology, Hawthorn, Victoria, Australia; 2 Sir Peter MacCallum Department of Oncology, The University of Melbourne, Parkville, Victoria, Australia; 3 Immune Signalling Laboratory, Peter MacCallum Cancer Centre, East Melbourne, Victoria, Australia; University of Tokyo, Japan

## Abstract

The quantification and analysis of molecular localization in living cells is increasingly important for elucidating biological pathways, and new methods are rapidly emerging. The quantification of cell polarity has generated much interest recently, and ratiometric analysis of fluorescence microscopy images provides one means to quantify cell polarity. However, detection of fluorescence, and the ratiometric measurement, is likely to be sensitive to acquisition settings and image processing parameters. Using imaging of EGFP-expressing cells and computer simulations of variations in fluorescence ratios, we characterized the dependence of ratiometric measurements on processing parameters. This analysis showed that image settings alter polarization measurements; and that clustered localization is more susceptible to artifacts than homogeneous localization. To correct for such inconsistencies, we developed and validated a method for choosing the most appropriate analysis settings, and for incorporating internal controls to ensure fidelity of polarity measurements. This approach is applicable to testing polarity in all cells where the axis of polarity is known.

## Introduction

Cell polarity is essential for the development and health of all multicellular organisms and controls diverse biological activities [1]–[4]. A facet of cell polarity that controls cell fate determination is Asymmetric Cell Division (ACD), a mechanism by which a dividing cell produces two daughter cells with different molecular composition, leading to the adoption of a different cellular fate [5], [6]. A role for ACD is now well established in cells of solid tissues, but its importance in lymphocyte development, function and disease is still controversial [7]–[12]. One of the major issues inhibiting elucidation of the role for ACD in lymphocytes is the difficulty in measuring asymmetry across these small, highly motile cells [13]. Although early studies of ACD focused upon examples where the asymmetry across the cell has been so obvious that subjective assessment could be used, other examples, such as lymphocyte ACD require finely tuned quantification.

Asymmetry in molecular localization is generally measured by fluorescent labeling of molecules within intact cells followed by fluorescence microscopic imaging. Fluorescent labeling might involve tagging of exogenously expressed proteins with genetically encoded fluorophores, or labeling of endogenous protein with fluorescently tagged antibodies. There are several approaches to measure polarity, some of which compare the geometric center of the cell with either the geometric centre of fluorescence or the brightest fluorescent pixel [14], [15]. An alternative approach, commonly used for measuring ACD, compares the total fluorescence from each half of the cell, often by deriving ratios of fluorescence in the two halves of the dividing cell [7], [8], [16], [17]. For this type of analysis, it is assumed that the ratios are proportional to the distribution of the molecules under investigation. The ratiometric approach has two advantages for ACD. First, the total fluorescence in each half is presumably more physiologically relevant than the other patterns of fluorescence within the cell, and should directly relate to the inheritance of those fluorescent molecules. Second, such the measurements can be continued beyond the point of cell division in time lapse imaging, making it more broadly useful for determining the functional consequence of ACD. Many variations of this approach have been implemented, such as comparing fluorescence along a line scan rather than using the total fluorescence, or measuring only nuclear asymmetry [18]. After deriving polarization measures in dividing cells, each event is then sometimes ascribed as Symmetric Cell Division (SCD) or ACD by arbitrarily assigning a cut-off value, with ratios above this arbitrary value considered asymmetric.

A ratiometric approach is only viable if the ratios that are derived from the fluorescent intensities are an accurate reflection of the ratios of protein in the two halves of the cell, and this has not previously been formally tested. Possible artifacts that might lead to inaccurate ratios include: the acquisition settings (such as detector gain, fluorescence excitation power, scanning parameters, fluorophore properties, and more), and intensity variations contributed from instrumental precision limitations such as signal-to-noise ratio (SNR) [19], [20]. Additionally, post-acquisition image processing such as background subtraction, spectral unmixing, and averaging algorithms can directly influence the fluorescence measurements in a nonlinear fashion [21]. To assess the reliability of quantitative fluorescence analysis, biologists can use internal controls, such as the parallel imaging of a molecule that is known to divide symmetrically [22]. Such an approach estimates the noise contributed from imaging artifacts such as uneven illumination or cell alignment (i.e. when the two halves of the cell are in different focal planes) [13]. However, because the fluorescence in the second channel is collected and processed differently to the channel of interest, this would not control for other acquisition and processing artifacts.

To the best of our knowledge there has been no report of how imaging settings and processing can affect the assessment of cell polarization. We recently showed that comparing fluorescence on the left half of the cell to that on the right half of the cell was useful in normalizing polarization of proteins during cell migration [23]. Here, we introduce a similar normalization approach to control for the effect on polarization ratios of non-biological factors such as image settings and post-acquisition analysis. To gain more insight into the effects of image processing on quantification of fluorescence ratios, and to develop new standardized approaches to correct for systematic differences that do not represent biological variation, we performed a comprehensive analysis using synthetic images. In this study, we focused on polarity during cell division, but the issues that we identify are common to all forms of cell polarity. We used both real data and computational simulations to identify pitfalls that currently prevent accurate analysis of cell polarity. A striking example is the large variance of calculated polarization ratios from a cell division that is known to be symmetric caused by altering the image threshold. To alleviate the problems inherent in polarity measurements, we introduced and validated a new corrective approach.

## Results and Discussion

### Polarization ratios are sensitive to image processing settings

**Figure 1:**
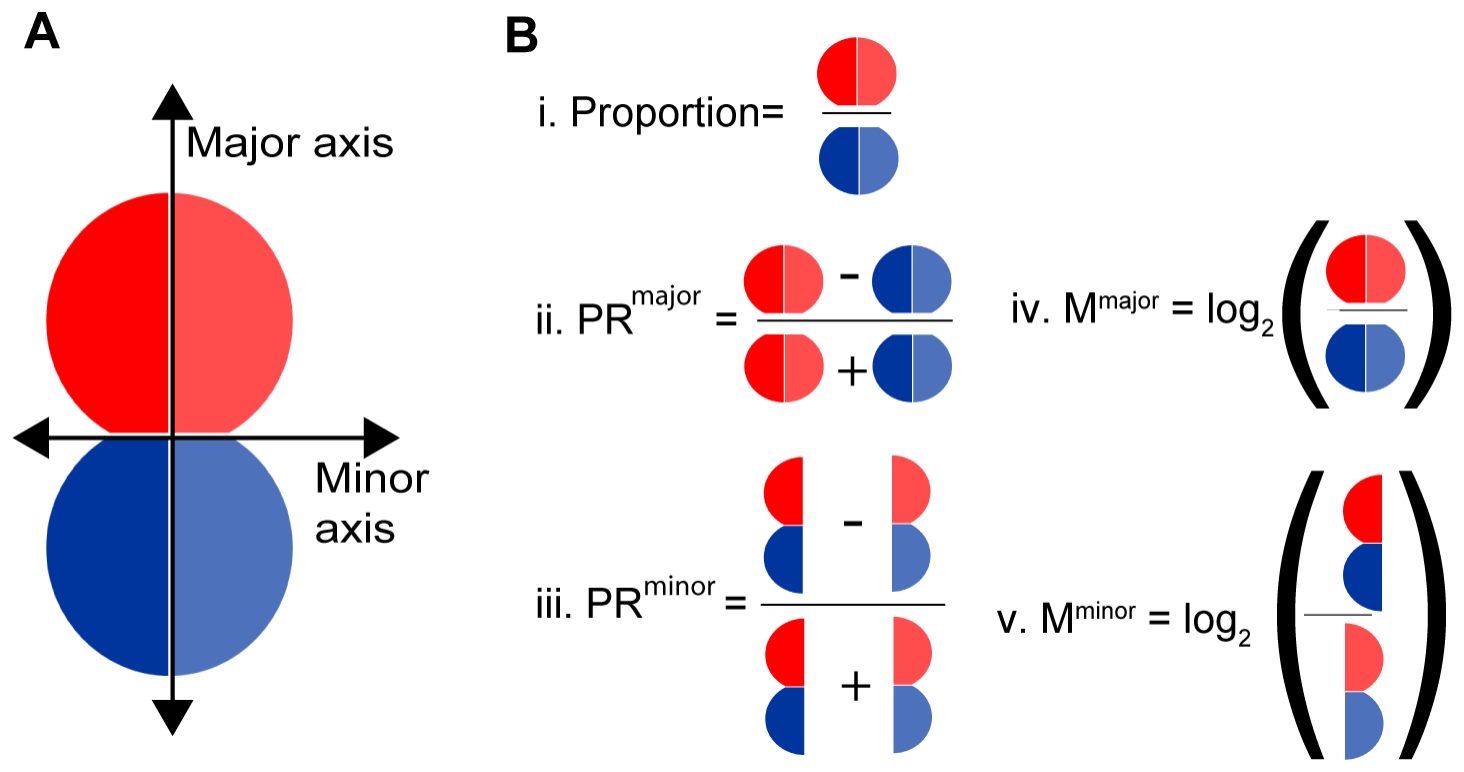
Axial subdivision for polarization analysis. (**A**) An approach for quantification of polarity. Black arrows represent the major and its perpendicular minor axis. Splitting the image into two allows a direct comparison of fluoresce intensity across the minor or major axes. The major axis is derived from the longest diameter of an ellipse that overlaps the cell. The minor axis is defined as the perpendicular to the major axis. Blue and red colors show the areas from which pixel intensities were collected, and represent the two halves of the cell that would, if the cell divided, become daughter 1 and 2. The left and right sides of the cells are bright and dim respectively. (**B**) Polarization ratios are extracted by integrating pixel intensities across the major or minor axis: i) Ratio along the major axis (using segments divided by the minor axis). ii) PR^major^: Normalized ratio along the major axis (across the minor axis). iii) PR^minor^: Normalized ratio along the minor axis (across the major axis).

First, we tested whether polarization ratio measurements are settings-dependent using a MLA-144 T lymphocyte cell line expressing freely diffusing Enhanced Green Fluorescent Protein (EGFP), which should always be distributed symmetrically. We focused on polarization during cell division, where morphology allows for straightforward designation of the two polarity axes. Cells were tracked, and the frames in which the shape of the dividing cell most closely represented the telophase stage were extracted for analysis. The major axis was used to assess polarization along the mitotic spindle (**Figure 1A**). Thresholding to remove background fluorescence is routinely used in image analysis, but from the analysis of cDNA microarrays it is known that intensity ratios are influenced by image processing [24] and the level of thresholding [25]. Therefore synthetic images were generated to explore the alterations in contrast that are frequently introduced during image acquisition or post acquisition processing, whereby pixels with intensities of a value less than the threshold value (between 0 and 90% of the value of the maximum pixel intensity of the cell) are either omitted [26] or set to 0 [27]. Similar effects can also be introduced experimentally during the image acquisition, for instance, by reducing the detection offset or the exposure time [28].

The threshold levels in the simulation vary between 0 to 90% of the value of the maximum pixel intensity of the cell (For additional data about the simulations see 'Materials and Methods', and **Text S1**, **Figure S1** and **Figure S2**). We tested the effect of removal of background fluorescence on two ratios that have previously been used to assess polarity in hematopoietic cells: a simple proportion between the integrated fluorescence of the two halves of the cell [7] (**Figure 1B** **i**) and a normalized Polarization Ratio (PR), calculated as the fluorescence difference between the two halves divided by the total fluorescence (**Figure 1B** **ii, iii**). In contrast to the ratio in Figure 1Bi, the PR accounts for potential artefacts that might be introduced where cells have heterogeneous expression levels, and has previously been used to calculate polarization ratios in cell-cell interactions [29], dividing [8] and migrating T cells [23]. PR^major^ describes polarization of relevance to ACD in these simulations (but could equally reflect polarization during migration), and PR^minor^ describes polarization along the axis perpendicular to the major axis.

**Figure 2:**
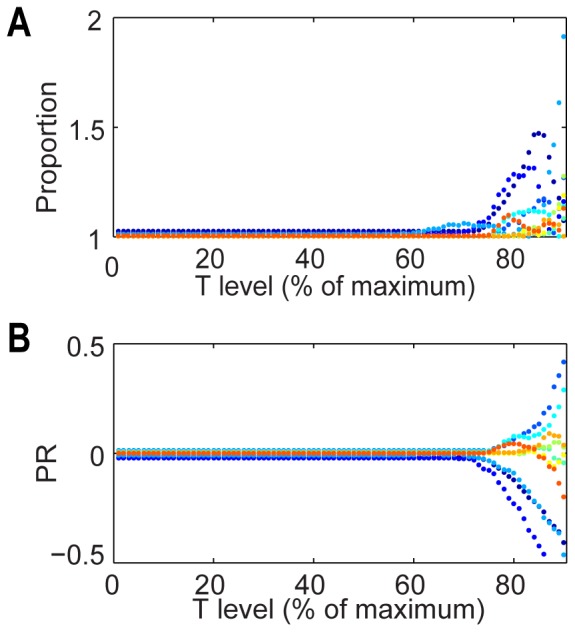
Ratio coefficients from experimental data are dependent on the threshold value. Fluorescence intensity ratios were extracted from images of dividing MLA cells expressing GFP, and for a random selection of 10 events, ratios plotted again threshold setting. Ratios were calculated using Proportion (**A**) and PR (**B**). Different colors represent different events.

Because GFP is uniformly distributed in cells, it is expected to give low PR. However, although ratios calculated in both ways are low (compatible with SCD) at threshold values of up to 65%, the ratios increase dramatically at threshold values greater than 65% for many of the cells (**Figure 2**). In extreme threshold values of T = 90%, some cells exhibited ratios larger than 1.5 (**Figure 2A**), a cut-off value that has been used for categorization of ACD [7], [30]. Similar trends were observed using the PR (**Figure 2B**). These data indicate that current quantification of polarization during division, which generally do not incorporate a systematic approach to background subtraction and contrast enhancement, run the risk of miscalculating polarization.

### Simulations indicate that the degree of clustering of fluorescence alters the effect of thresholding

**Figure 3:**
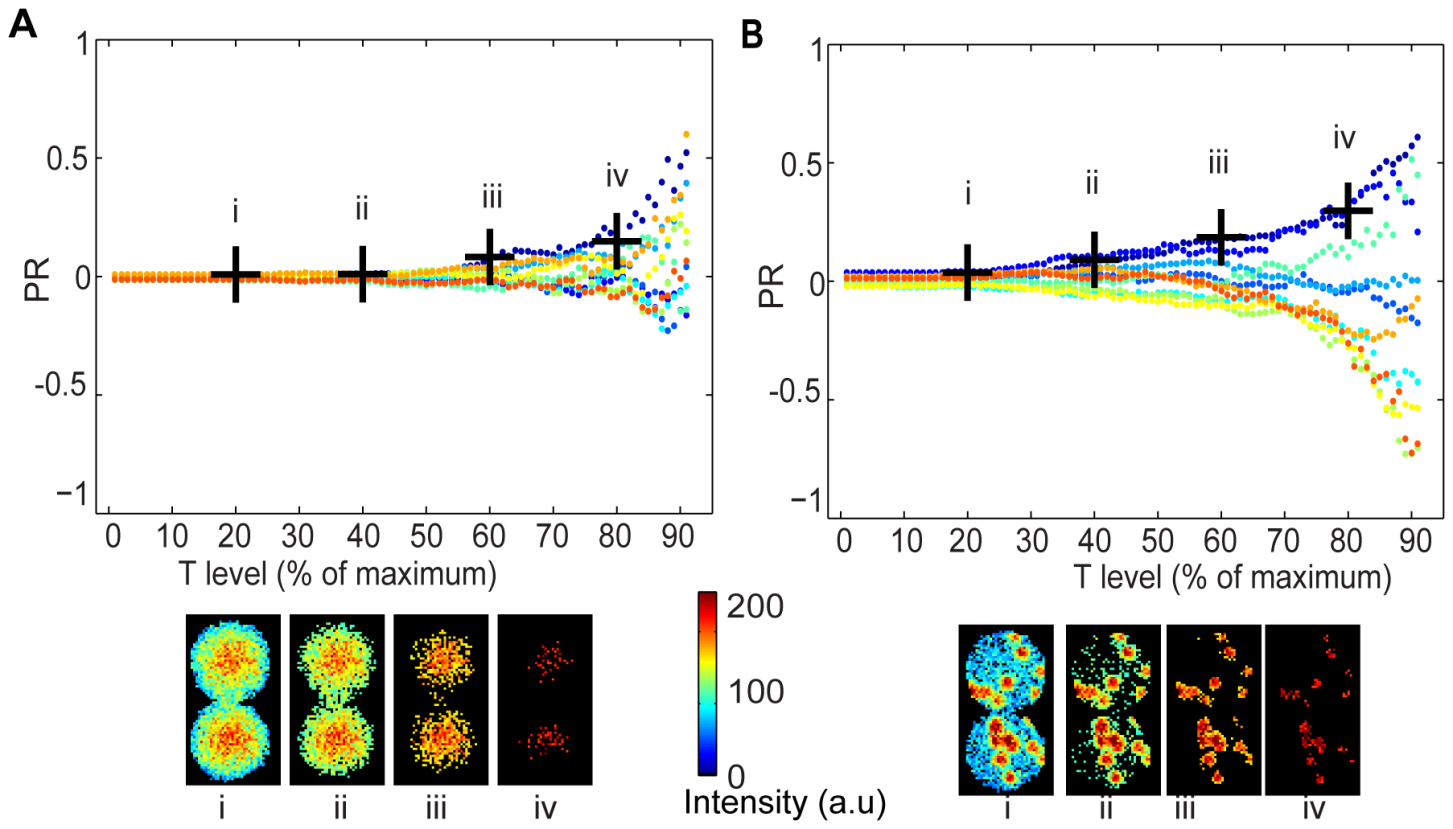
Ratio coefficients from synthetic data are dependent on the threshold value. 10 images of cells simulated to have symmetric fluorescence partitioning with non-clustered (**A**) and clustered localization (**B**) were simulated to quantify the effect of background subtraction on ratio coefficients. The horizontal axis represents the Threshold value. The vertical axis represents the PR. The possible range varies between -1 to 1, where 0 is maximum symmetry and high absolute ratios indicate maximum asymmetry. Different colors represent different simulated divisions. Black crosses represent the ratio and T value of illustrative images below. Input for simulation: θ = 0, R = 22 pixels, number of clusters in both D1 and D2 was 20.

In addition to the impact of acquisition and analysis settings as discussed above, many intracellular molecules are not distributed as homogeneously as EGFP, which might lead to further artifacts. To assess this, we simulated cell divisions with fluorescence in either clustered (representing protein aggregates, nanoparticles, or endosomal proteins [31], [32]) or non-clustered (representing diffusely distributed proteins such as EGFP) patterns, each with both symmetric and asymmetric distribution. PR values for 10 simulated symmetric divisions of non-clustered fluorescence (**Figure 3A**) over different threshold values showed a similar pattern to the EGFP-expressing cells (Figure 2B), with threshold levels of greater than 80% (Figure 3A.iv), yielding high PR values that could be falsely interpreted as ACD. Simulations of symmetrically dividing cells with clustered fluorescence (**Figure 3B**) showed a further increase in sensitivity to thresholding, with threshold levels over 40% yielding PR values of 0.1. At extreme levels of thresholding (of more than 80%, Figure 3B.iv) all pixels that remained were from the original clusters. At this level of thresholding, PR values could exceed 0.3 (corresponding to an absolute ratio of 1∶1.5), despite the fact that the division was symmetric. Thus, the simulations demonstrate an influence of image processing settings on the measured ratios that is similar to that observed with the EGFP-expressing cells, indicating their suitability for further analysis. Furthermore, the simulations indicate that cells with clustered fluorescence are still more susceptible to artifacts, further highlighting the need for a systematic method for image processing and background subtraction.

### Calculation of the polarity across the minor axis provides a useful control to assess noise and to normalize calculations of asymmetry

**Figure 4:**
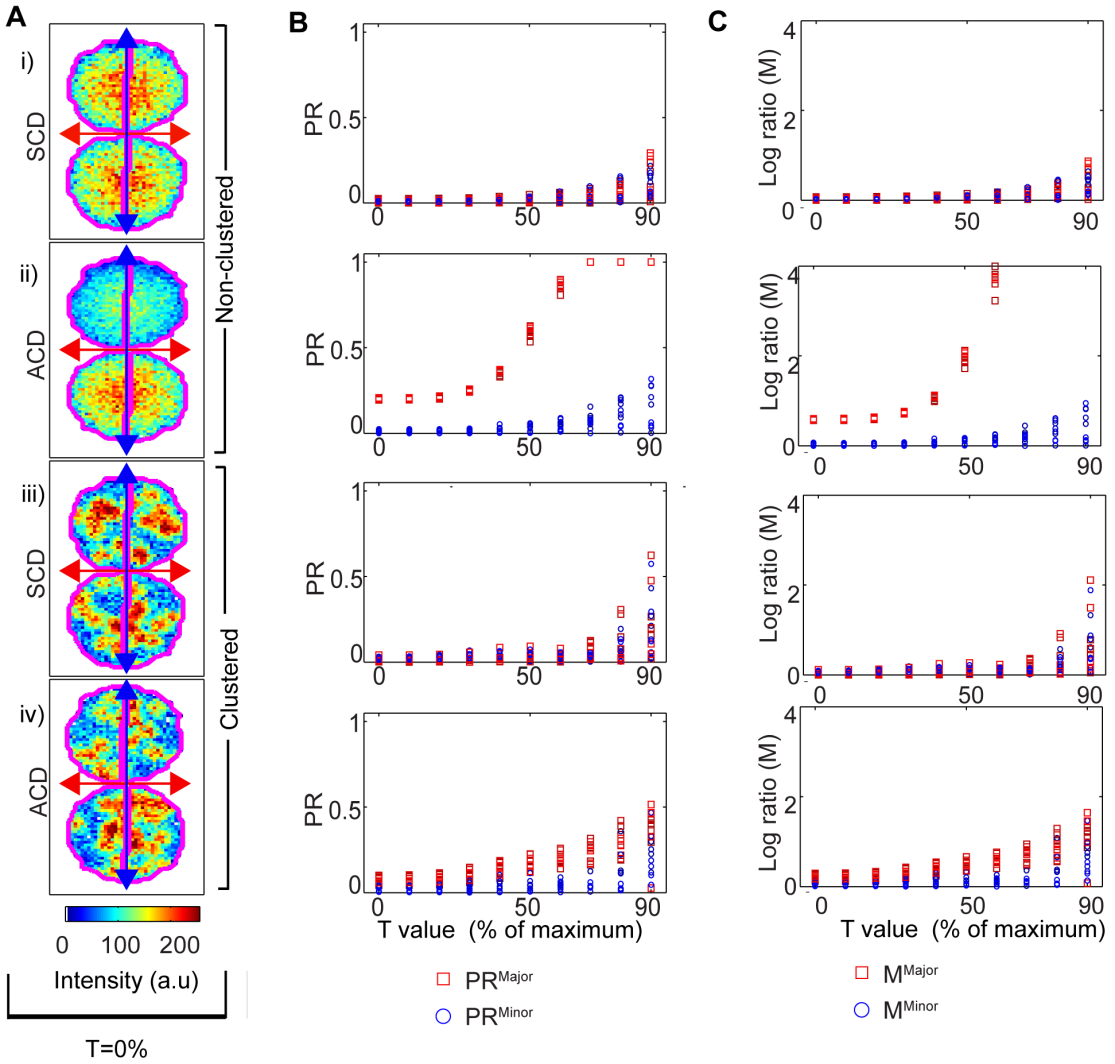
PR^major^ and PR^minor^ are differentially affected by thresholding and clustering. Cells were simulated to be: i) symmetric non-clustered; ii) asymmetric non-clustered; iii) symmetric clustered; iv) asymmetric clustered. (**A**) Examples of simulated cells and approach to hemisphere separation. Blue and red lines describe the major and minor axes respectively, and the magenta contour shows the separation that gave an equal number of pixels to each hemisphere (slightly shifted from the major axis). (**B**) PR^major^ and PR^minor^ values for 10 simulated cells were plotted against T value (**C**) M^major^ and M^minor^ values for 10 simulated cells were plotted against T value. Note that in the asymmetric cells, some fluorescence values were reduced to 0 for the higher threshold settings, causing misleading values of 1 in (B) and infinite (unplottable) values in (C). Input for simulations: θ = 0, R = 22 pixels, number of clusters in D1 and D2 was 20 in the symmetric and 20 and 30 in asymmetric

A unique characteristic of most polarized cells was previously shown to provide an opportunity for improved analysis [23]. Namely, that true asymmetry should be evident across one axis, but should generally not occur across the perpendicular axis (Figure 1A). In contrast, asymmetry that is caused by artifacts in the image acquisition or processing should be evident across both axes. The principle of our method is that settings applied during and after image acquisition will cause similar skewing of the ratio across both axes. The ratio across the minor axis can therefore be used to estimate the noise in the PR measurements. To assess whether this approach might enable a systematic method to select image processing settings, and to determine the influence of background removal on minor and major ratios, 10 cells were simulated (representative cells are shown in **Figure 4A** and **Figure S3**) for: i) symmetric non-clustered; ii) asymmetric non-clustered; iii) symmetric clustered; iv) asymmetric clustered cell, and the PR^major^ and PR^minor^ were compared (**Figure 4B**). Symmetrically dividing cells exhibited low PR values for low threshold settings, and the PR increased slightly at higher settings. Importantly, the increase in PR^major^ at high segmentation settings was similar to the increase in PR^minor^, indicating that these polarization measurements reflected artifacts in image processing rather than genuine asymmetry along the major axis. The simulations of asymmetrically dividing cells showed a strikingly different pattern, where the PR^major^ and PR^major^ were clearly different from each other. In non-clustered fluorescence (Figure 4ii), PR^minor^ was low (below 0.05) at low threshold settings, and increased slightly at higher threshold settings (to a maximum of 0.2). In contrast, the PR^major^ hovered around 0.2 for low threshold values but increased dramatically at high threshold values, plateauing at 1.0 by 60% thresholding (where threshholding can lead to aberrant values, rendering the data meaningless). These data indicate that the pattern of PR^minor^ response to threshold settings provides an opportunity to set the appropriate conditions for measurement of PR, and can be used to objectively ascribe an appropriate threshold value for analysis of the PR^major^. For instance, the appropriate threshold value for analysis could be defined as the value just below that at which PR^minor^ increases by 10% above the baseline (50-60% in this instance). Simulated cells in which the fluorescence was clustered showed a similar, although more noisy, trend, with low PR^major^ and PR^minor^ values across the thresholding range for cells with symmetric distribution of clustered molecules. However, the PR^major^ was clearly different from PR^minor^ at higher threshold levels for asymmetric distribution of clustered molecules. In microarray analysis, the log ratio has proved to be as good as or better than the PR value for comparing pairs of fluorescent values, thus we determined whether this approach might also be useful for polarity measurements by deriving log ratios across the major and minor axis (**Figure 4C**). The log ratio also provided good discrimination between symmetry and asymmetry, which again was highly dependent upon the thresholding. The log ratio approach did not present any obvious advantages over the PR, and since the PR is more established in cell polarity measurements, we focused on PR for the remainder of this study. Together, these data confirm that the PR^minor^ could be used to assess the reliability of ratiometric measurements and to select an appropriate thresholding setting for analysis of PR^major^.

### Comparing PR^major^ and PR^minor^ demonstrates the effects of clustered fluorescence

**Figure 5:**
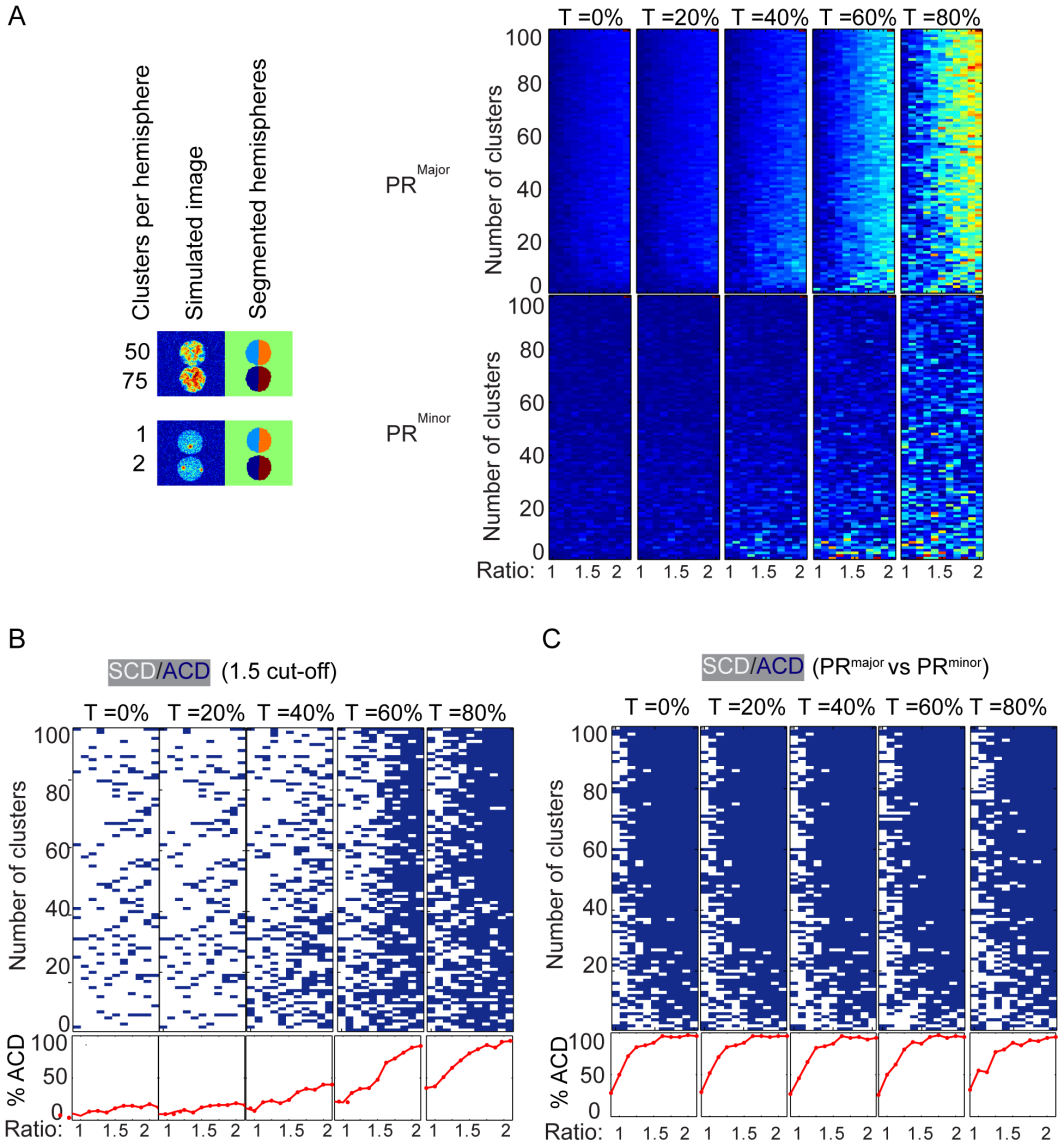
The effect of cluster number on the accuracy of PR measurements. Divisions were simulated to have increasing numbers of clusters ranged from 1 to 100 in increments of 1 for one of the daughter cells. The number of clusters in the second daughter was the number of clusters in daughter 1 multiplied with its corresponding ratio. Ratios vary from1 to 2 with increments of 0.1, and were calculated under 0%, 20%, 40%, 60%, and 80% threshold. (**A**) The PR^major^ and PR^minor^ for each event are shown in heat maps, where the PR ranging from 0 to 1 are represented in "jet" colors (blue to red). The ratios were binarized using (**B**) cut-off value of 1.5 or (**C**) by equation 5. Blue pixels represent events in which PR^major^ was larger than PR^minor^; white pixels represent events in which PR^major^ was smaller than PR^minor^.

Having shown that a clustered pattern of fluorescence increases the PR (Figure 4), we used the differences between PR^major^ and PR^minor^ to assess the degree of clustering for which ACD could be reliably assessed. We simulated divisions of cells containing from 1 and 100 clusters (increments of 1), and with fold-differences in the number of clusters from 1 (symmetric along the major axis) to 2 (asymmetric along the major axis) with increments of 0.1 (**Figure 5**, two examples shown on left). PR^major^ and PR^minor^ were displayed in heat maps (**Figure 5A** right hand side), calculated under 0%, 20%, 40%, 60%, and 80% threshold values. Again, increasing threshold levels resulted in an increase in both PR^major^ and PR^minor^, but particularly promoted an increase in PR^major^. As expected, at high numbers of clusters, the data appeared similar to the unclustered analysis above: PR^minor^ was aberrantly high at thresholds of 80%, but at T = 60%, PR^minor^ was reasonably low and PR^major^ showed a good dynamic range. At T = 60%, PR^minor^ was reasonably low even as the cluster number diminished to approximately 8–10 clusters per cell. However, below 8–10 clusters per cell, PR^minor^ was aberrantly high, as was the PR^major^ for simulations in which the symmetry across the major axis was close to 1, which should not have yielded high values. These data suggest that clustering can still yield reliable polarity ratios, as long as the number of clusters exceeds 8–10. Evidently, different data sets might be more or less affected by cluster number, and we were conservative in simulating only weak polarity (2 fold differences across the major axis), so the analysis of more extensive polarity should be even more robust. Nonetheless, these data both demonstrate that clustered fluorescence can yield robust polarity analyses if care is taken to ensure the best settings, and indicate the value of PR^minor^ to assess the noise across a non-polarized axis and ensure appropriate quantification.

### Assessing the value of binarization in the analysis of polarity

In many biological situations, a population of cells can comprise both symmetric and asymmetric divisions [33], in addition to divisions that were imaged or processed at wrong settings. In such cases, it is desirable to measure the degree of asymmetry of both the population and of individual cells, and, in some instances, also to binarize such data and to designate each cell as symmetric or asymmetric. Binarization has previously been achieved by assigning a cut-off value and binning all events above the cut-off as polarized, and all events below the cut-off as non-polarized [7]. Although convenient, such an approach clearly has the potential to introduce errors, as it does not account for the distribution of polarization ratios, the potential overlap of ratios amongst two subpopulations, or the errors in individual events. We utilized the results of Figure 5A as ground truth data to formally assess the accuracy of the cut-off approach, and binarized as ACD or SCD using a cut-off of 1.5 (as has previously been used, [7], [9], [30]) (**Figure 5B**). The number of events ascribed as ACD increased as the ratio increased (quantified in the histograms below each heat map), and that this was most evident at 60% thresholding (previously shown to be the most appropriate setting). However, the data was noisy, with many blue events (ACD) in the left columns (symmetry), and many white events (SCD) in the right columns (asymmetry). For this simulation, at the most appropriate threshold (60%) approximately 28% of the cells that were simulated to have a ratio of 1.0–1.3 were scored as ACD (i.e. 28% false positives), and 82% of cells that were simulated to have a ratio of 1.7–2.0 were scored as ACD (i.e. 18% false negatives).

This analysis indicates that simply deriving a ratio and ascribing a cut-off can lead to error in the designation of divisions as ACD or SCD, and that there is no value that would effectively discriminate between high and low polarization ratios. We then assessed whether PR^minor^ might enable an alternative approach to binarization of the data. The analyses described above use PR^minor^ to assess the noise in populations of cells and to optimise processing settings, but PR^minor^ of individual cells also has potential value in the analysis of polarization of the population. We therefore used the data set in Figure 5A to determine the value of binarizing by simply comparing PR^major^ with PR^minor^. ACD was ascribed to cells in which PR^major^ was greater than PR^minor^, and SCD was ascribed to cells in which PR^major^ was less than or equal to PR^minor^ (**Figure 5C**). At all thresholding settings the number of events ascribed as ACD increased with increasing polarity, but as with the cut-off approach above, false positives (1∶1 simulations ascribed as ACD) occurred even under optimal conditions (60% thresholding, high numbers of clusters). The number of false negatives (2∶1 simulations ascribed as SCD) was low across all thresholding settings, and these were only evident for simulations with low numbers of clusters. Compared with the cut-off approach (Figure 5B), at the 60% thresholding levels, there were more false positives (54%), but fewer false negatives (5%) and the binarization was far less dependent upon thresholding than the cut-off approach. Interestingly, visual inspection of binarization plots indicated that the accuracy of binarization was much more dependent on clustering for the PR^major^ versus PR^minor^ approach than for the cut-off approach (presumably, because artifacts due to clustering can have twice the impact). These data indicate that binarization using either of these methods can be useful for comparing between two populations, that this approach is more appropriate for non-clustered data, and that events that are ascribed as SCD are most likely truly symmetric. Most importantly, these data indicate that binarization is not a reliable indicator of the number of truly asymmetric events, and the designation of an event as symmetric or asymmetric requires a more case-by-case approach.

### Methods to incorporate PR^minor^ in the assessment of polarity

**Figure 6:**
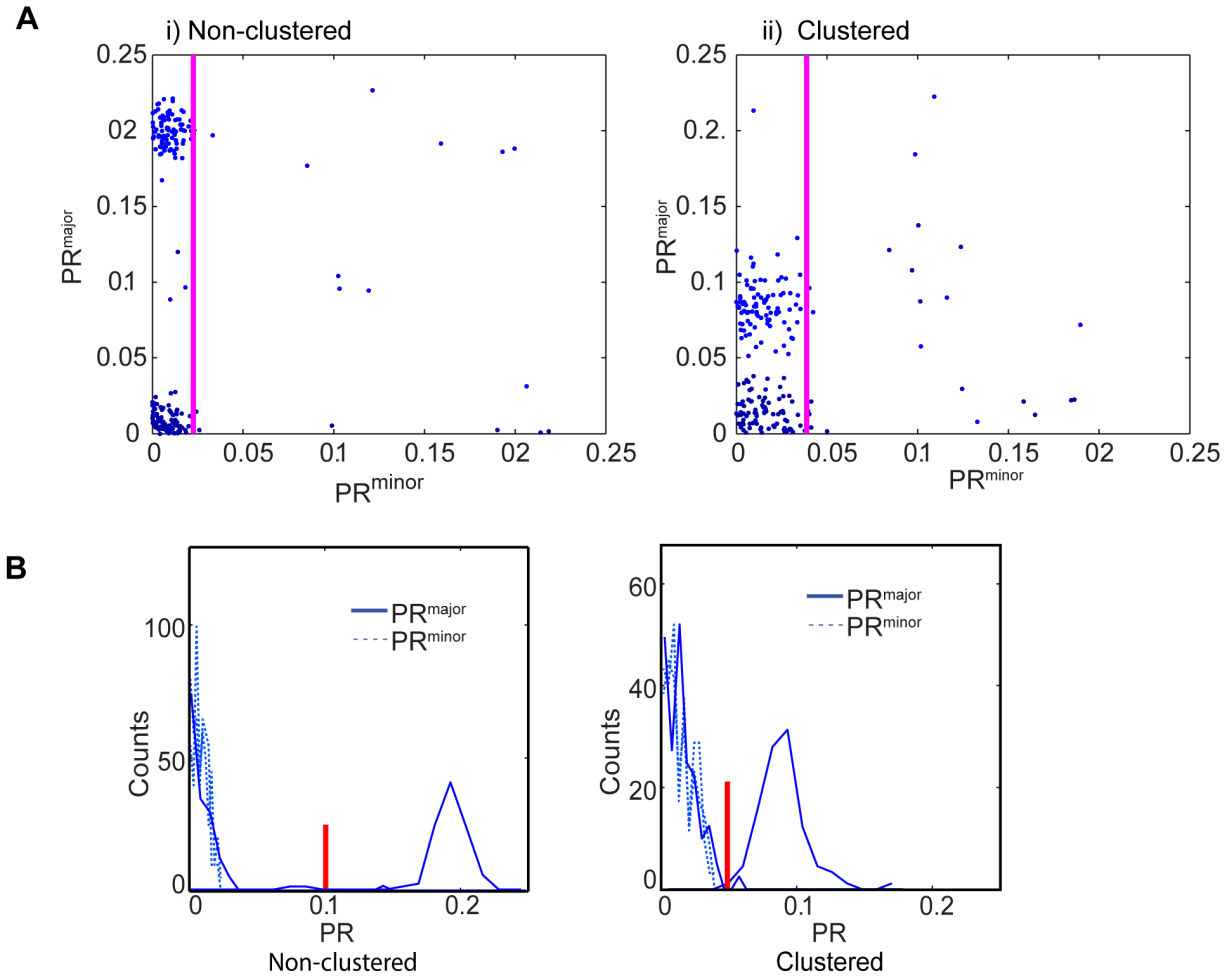
Gating based on PR^minor^ in simulated data. Cell divisions were simulated with ratio 1 (blue color) or 1.5 (red color), 100 divisions for each, with 10% of out of focus "bad data" and PR^major^ (y-axis) was plotted against PR^minor^ (x-axis) (**A**) for non-clustered (i) and clustered (ii) fluorescence. The magenta line show the gating border that to removes 10% outliers of the minor axis polarization, to remove "bad data". PR^major^ of the gated events were plotted as a (**B**). Input for simulations: θ was chosen randomly varying from 0 to 90 degrees, distribution of parental radius and total intensity were selected randomly from real distribution from real data, number of clusters in one of the daughter cells was 20 to 100, and the number of clusters in the other daughter cell was multiplied in the simulated ratio giving possible range from 30 to 150.

The simulations in Figure 5A indicate that, even after careful selection of thresholding values, PR^major^ is still noisy, particularly for clustered fluorescence. To assess whether PR^minor^ could be used to improve the analysis, we adopted a plot of PR^major^ versus PR^minor^ as used previously to explore the relationship between PR values [23]. We next assessed whether PR^minor^ could also be used to remove aberrant events and so improve the quality of the analysis, by comparing the PR^major^ vs. PR^minor^ plots from five different thresholding values (**Figure 6A**). These simulations incorporated ground truth "bad data" to assess the value of this approach: Each division was simulated to have the probability of p = 0.025 that one daughter cell was out-of-focus, one side of the two cells was out-of-focus, one side of one of daughter was out-of-focus, or only one side of one of daughter was in focus. In total, 10% of the data is expected to contain an out-of-focus artifact. The simulations were designed to represent a mix of symmetric and asymmetric events, with minimal events of intermediate polarization. As in flow cytometric analysis, visual inspection of the PR^major^ vs. PR^minor^ plots provides much information: (i) a small number of outliers were evident, which can then be discarded from the analysis, (ii) a majority of events with low PR^minor^ were clearly suitable for analysis, and (iii) a clear distribution into two populations of PR^major^, representing symmetric and asymmetric divisions could be seen. The "bad data" was removed by gating out the top 10% of PR^minor^ values, and, PR^major^ and PR^minor^ were represented as histograms (**Figure 6B**). In this instance, the distribution of the histogram allows clear discrimination between the two populations, making allocation into ACD and SCD populations both facile and valuable. In most real scenarios, there would probably not be a clear discrimination between the two subpopulations, and binning into ACD and SCD would likely require approaches such as those described above, with the accompanying issues of accuracy. These data illustrate that the comparison of PR^major^ and PR^minor^ on individual events using a two-dimensional scatter plot provides and effective means to assess the quality of the data, gate out artefacts, and assess the distribution of polarity across the population. Conversion of the data to one-dimensional histograms allows for comparison across populations, and provides the basis for whether, and how, each event might be binned as ACD or SCD.

### Sensitivity test from simulations

**Figure 7:**
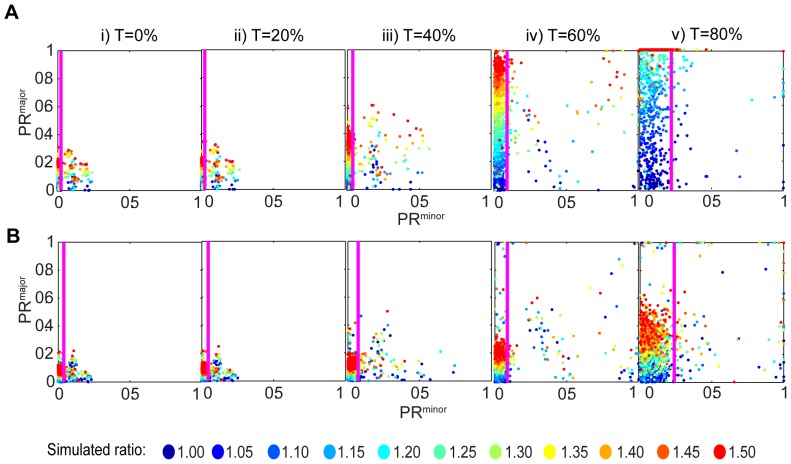
Sensitivity test from simulations. Cell divisions were simulated in increasing ratios from 1 to 1.5 with even increments of 0.05, giving n = 1100 divisions in total. PR^major^ was plotted against PR^minor^ for non-clustered (**A**) and clustered (**B**) data. Data is showed as major/minor plot, under a range of thresholds from T = 0%, to T = 80% in increments of 20%. The magenta line shows gating exclusion of 10% of data with the highest PR^minor^. The gate shifts right as T value increase. The colours in the figure legend represent different ratios and corresponding to the ratio colour of each data dot. Input for simulations: θ was chosen randomly varying from 0 to 90 degrees, distribution of parental radius and total intensity were selected randomly from a real distribution from real data, number of clusters in one of the daughter cells was 20 to 100, and the number of clusters in the other daughter cell was multiplied in the simulated ratio giving a possible range from 30 to 150.

The above analysis illustrates the difficulty of designation of events as ACD or SCD. An ideal experimental situation would contain known symmetric and asymmetric controls for comparisons with the test samples, and provide a basis for determining the best binarization approach, but such controls are not always available. The analysis performed in Figure 6 assumes that there are two populations that can be relatively easily separated to two clusters. However, in some cases the classification can be much harder. To study the value of our approach in samples without the clearly defined two populations described in Figure 6, we simulated a heterogeneous population of 1100 divisions in which the proportional ratio between the two daughter cells, varied from 1 to 1.5 fold with 100 cells representing each increment of 0.05. Scatter plots of PR^major^ and PR^minor^ for each simulated division at threshold values of 0%, 20%, 40%, 60%, and 80% (**Figure 7A and B** for non-clustered and clustered respectively) showed that 60% thresholding provided the most suitable dynamic range for PR^major^. The gate excluded all events in the top 10% of PR^minor^ values (right of the pink line). For non-clustered simulations, color coding (each colour represents simulations within a 0.05 window of ratios) indicates that the gated events generally yielded appropriate PR^major^ values. These data indicate that gating removed some mid-range events that would otherwise have been allocated an inappropriate PR^major^ (out-of-focus events were not simulated in this experiment, but should have also been reduced by this process). Gating out the top 10% of PR^minor^ was particularly important for the analysis of clustered events, where PR^major^ was appropriately distributed according to colour for the low PR^minor^ events, but inappropriately distributed in the high PR^minor^ events (see histograms in **Figure S4**). Combined with the analysis of out-of-focus events in Figure 6, these data provide strong support for the value of plotting PR^major^ against PR^minor^ to exclude aberrant data. In addition, the finding that PR values distributed according to original input ratios (as illustrated by the color distribution in Figure 7), indicates that this approach can discriminate between increments of polarity, providing greater value than a mere allocation as asymmetric or symmetric.

### Strategy for polarity analysis

**Figure 8:**
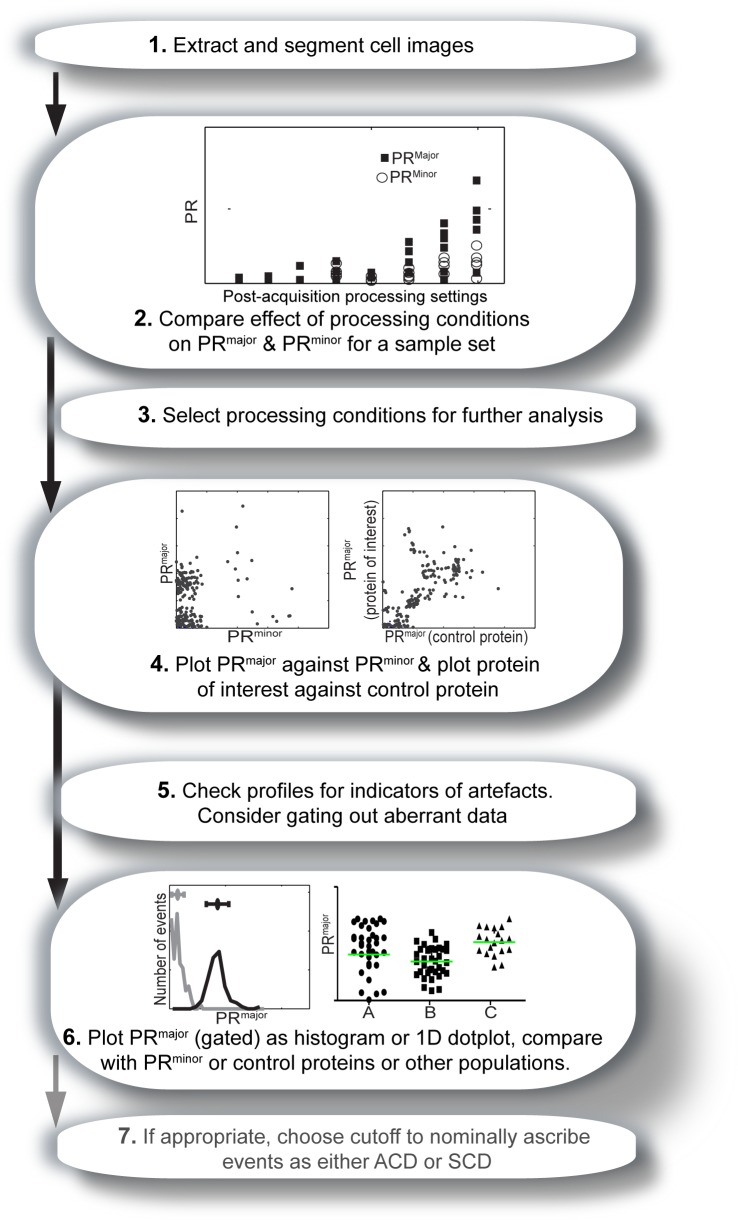
Suggested workflow for optimal analysis of ACD. A method of analysis that avoids some pitfalls of polarity measurement as illustrated in this study begins with (**1**) extraction and segmentation of images, including demarcation of major and minor axes (either using the long axis as demonstrated here, or alternative strategies). (**2**) A randomly selected sample set of the data should be used to plot PR^major^ and PR^minor^ against a range of processing settings, and used to (**3**) determine the optimal processing settings that avoid artificially high PR values (as indicated by PR^minor^ analysis) but provide good dynamic range of PR^major^(see the discussion of Fig 4 for an illustration of this process) (**4**) The optimal processing settings are used to plot PR^major^ against PR^minor^ for the entire population. (**5**) PR^major^ vs. PR^minor^ is utilized for exploration of the quality of the data. Firstly, assuming that polarization occurs only along one axis, the two parameters should be independent of each other and this can be evaluated from the plot both visually (as is common in flow cytometric analysis where correlations can indicate errors in cross-spectral compensation) and by regression analysis. If the plots are still linked to the original data, any outliers can be readily examined to determine possible causes of error. For instance, using an interface such as provided by the TACTICS Toolbox [23], clicking on the dots can bring up the specific frame or movie associated with that data point and possible exclusion of aberrant data such as problems with the focus. Secondly, gating for cells with low PR^minor^ values on the plots enables exclusion of noisy data and simultaneous assessment of the extent, range and variance of PR^major^. (**6**) The gated PR^major^ can then be plotted as a histogram or scatter plot, enabling comparison with control data or between test populations. These plots represent an endpoint of the analysis, but can also be used to determine whether additional values such as mean or median PR, range, variance or proportion in different PR values would be informative and could be extracted from the data. (**7**)(**Optional**) Depending upon the quality of the data and the goals of the analysis, binarization of the events into ACD and SCD could be achieved by either cut-off or comparison of PR^major^ and PR^minor^ values as described in Figure 5.

Normalization of polarization ratios against control fluorophores or the minor axis, allows for an objective assessment of the degree of asymmetry. This brings us closer to the ultimate goal of developing a means to determine whether ACD occurs in a population or in individual cells, even when ground truth data such as the degree of asymmetry in biologically relevant ACD events is not available. Although this approach was developed specifically for the analysis of ACD, it also provides a guide for the analysis of all forms of polarity in lymphocytes and potentially in other cell types. A proposed workflow for one approach to the analysis of polarization is described in **Figure 8**. In this approach, plots of PR^major^ and PR^minor^ are generated to compare any post-processing alterations under consideration (such as the variations in segmentation settings used in this study), and to select settings that do not increase the PR^minor^, but provide good dynamic range for PR^major^. Ideally, a random subset of the data would be analyzed to assess variation without biasing towards the final outcome would be used for this assessment (for instance, histograms plotting PR^major^ and PR^minor^ against segmentation for 10 randomly selected events. The same process can be undertaken for fluorescence of a control protein, if available, as different processing settings might be more appropriate [23]. Having selected the most appropriate settings for each fluorescent colour, all the data can be displayed in a two-dimensional scatter plot of PR^major^ versus PR^minor^, and also PR^major^ for the fluorescent molecule of interest plotted against PR^major^ for the control protein. These plots can be scrutinized for indications of any possible problems with the data. For instance, if the two different fluorescent signals correlate, this might suggest that there is spectral bleed-through between the two fluorescent channels that needs to be corrected. Out-of-focus events might also be identified. Ideally, events that are inappropriately high for either PR^minor^ or for the control protein PR^major^ would be assessed to determine the cause of the issue, and depending upon the distribution of the data, and these events can be gated out for subsequent analysis of PR^major^ of the control protein. This data can then be presented as a histogram (or one-dimensional scatter plot depending upon the sample size), and can be plotted alongside controls such as the PR^minor^ of the protein of interest, and the PR^major^ of the control protein. As with flow cytometry, the question being explored will then determine whether the histograms are used to comparison between populations, to derive mean or median polarization ratios, or to further subdivide the populations as polarized or not polarized. Additionally, it can be used to consider possible differences in other aspects and measures between two daughter cells such as size, migration patterns, and more. TACTICS software (http://tactics-toolbox.com/) [34]provides one valuable means to follow this process, and conversion of microscopic data to a format readable by standard flow cytometric data [35] would presumably provide another. This strategy is applicable to studies of polarization during cell division as exemplified here (see **Figure S5**), cell migration [23], and presumably other forms of polarity such as immunological synapse formation and apicobasal or planar cell polarity [3], [4].

There are several ways in which quantitative data can be verified to ensure that it genuinely reflects the polarity of the cell, the approach we suggest here provides one method, but each experiment will provide different opportunities for validation. For instance, in time lapse microscopy, measuring fluorescence of the daughter cells would allow assessment of whether a polarity ratio correlates with asymmetric inheritance. Controls that are expected to alter polarity can also be informative regarding the validity of the measurements. The use of controls such as PR^minor^, and the application of gating to remove aberrant events, approximates more closely the rigour in analysis that is now standard in flow cytometry. Multiparametric comparisons, quality control and interactive probing of the data have proven extremely valuable for flow cytometry, and applying such approaches to the wealth of contextual information available in microscopy experiments should dramatically enrich our understanding of biology. In summary, our results demonstrate that currently used approaches to the quantification of biology can be misleading, but that the use of appropriate controls and analytical approaches can allow for reliable measurements.

## Materials and Methods

### Computational platform

Generation and analysis of both synthetic and real images was achieved using MATLAB R2012b version 8.0 (the MathWorks, Inc., Natick, MA, USA) with the Image Processing Toolbox. Calculations were performed on an HP Z400 workstation equipped with a 3.3 GHz Intel Xeon W3580 Quad processor and 16 GB of RAM working under a Windows7 64-bit operation system.

### Generation of synthetic images

Binary cell images were generated using a simplistic model of dividing cells as explained in **Text S1**. Our simulations exemplify non-clustered molecules, such as GFP in addition to clustered molecules such as nanoparticles [18], [36], protein aggregates, [37] or mRNA [15] that are characterized by speckled density spots. The texture of non-clustered, freely distributed fluorescence was generated using MATLAB implementation of Perlin texture created by Antti Lehmussola [38]. The MATLAB function spotmaker.m (written by Tristan Ursell 2012, downloaded from-http://www.mathworks.com/ MATLABcentral/fileexchange/36026-create-a-simulated-image-of-diffraction-limited-spots-with-noise) was used to generate clustered localization. Multiple clusters were simulated as diffraction-limited spots with noise. The initial position of the spots was randomly distributed, and for sequential frames the location was randomly moved in the x and y direction to simulate Brownian motion. To mimic the fact that fluorescent images are frequently derived from a projection of the 3-dimensional volume of the cells, we used a 2-dimensional radial intensity distribution with exponential slope over the polygon from its center:



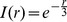
(Equation1)Where r is the pixel coordinates around the origin. Finally, further imaging effects were added to give more realistic characteristics. To simulate the Gaussian point spread function (PSF) typical of confocal fluorescent images, the clustered images were filtered using the MATLAB function imfilter with a Gaussian filter. To simulate a blurred effect for the non-clustered expression we used a disk filter. Both filters were generated by the MATLAB fspecial function. Realistic simulated data that contain some degree of noise was generated with skewed polarization ratios. For this aim, MATLAB imfilter function was utilized to convolve a Gaussian filter only to the part of cell that is out of the focal plane. To simulate white noise and detection response, Gaussian white was added using the MATLAB function, imnoise. Finally, each division was aligned along the major axis and the daughter cells categorized as daughter 1 for the top and 2 for the bottom cell, positive for right side and negative for left side. However, this labeling was only used to separate between the cells and had no effect on the analysis as the cells were randomly flipped to remove any potential systematic bias. The MATLAB source-code of TACTICS, and the simulations are available online at http://tactics-toolbox.com.

### Cell paddocks and cell culture

Cells were cultured in microfabricated grids (paddocks) for cellular studies in vitro [39]. The cell paddocks were made of transparent biocompatible polymer PolydImethylsiloxsane (PDMS) with dimensions of 125×125×45 micron and with well-defined vertical sidewalls and a transparent base. Cell paddocks were placed into a well of an 8 well chamber slide (LAB-TEK II, NUNC) sterilized with 100% EtOH and UV light, and rinsed with media prior to use. MLA-144 T cells expressing GFP were cultured at 37°C, 10% CO2 in Dulbecco's Minimal Essential Medium (SECF) supplemented with 10% (v/v) fetal calf serum, L-glutamine (1 mM) and 100 ng/mL penicillin/streptomycin.

### Time-lapse microscopy

Time-lapse images were obtained with IX71 inverted microscope (Olympus, Tokyo, Japan) equipped with a Nipkow disk-type confocal unit (Yokogawa CSU22, Tokyo, Japan) and Electron Multiplying Charge-Coupled Device (EMCCD) Andor camera (Model: iXon EM+885, Belfast, Northern Ireland). Images were acquired in both DIC and green channels using a 20x air objective 0.45NA, which corresponded to a pixel size of 0.33 µm×0. 33 µm. The working distance was 6.6–7.8 mm. Exposure time was 600 ms for green and 100 ms for DIC. Multiple stage positions were captured (controlled by MetaMorph software version 7.7.11.0) with a sampling rate of 1 minute for 1–24 hrs, and were saved as 8-bit two-dimensional arrays (1002×1004 pixels).

### Processing of time-lapse data

Divisions were split by minor and major axis. Pixels of the two corresponding sides of the cells were copied to reconstruct two new images, whereas each image represents one half of the cell. To define the major axis, a line-based Bresenham algorithm [40] was stretched across a defined axis to split the cell image through the cell center into the two opposite pixels located on the cell perimeter. The minor axis is perpendicular to and passes through the midpoint of the major axis. When cells are close to each other it is impossible to accurately separate the pixels. Therefore, automated selection for splitting was applied by fitting the cell shape and area to a circle to find the angle of overlap θ. When θ was smaller than 37 degrees, instead of trying to match from which daughter the overlapping pixels belong, the splitting was made so that an even number of pixels was contributed from each daughter. Since the use of subtraction techniques such as threshold has been shown to change the apparent size measurements [41], cell borders were kept constant and the initial detected borders in (T = 0) were used when screening the effect of T value on PR.

### Calculation of Polarization Ratio (PR)

Throughout this study the Polarization Ratio (PR) values were calculated as the fluorescence difference between the two halves using the equation: 
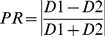
(Equation2)where D1 and D2 are the two halves of the cell, which can be either the mean or integrated pixel intensity, depending upon the experiment. To calculate the PR^major^ the two halves are daughter 1 and daughter 2 (Figure 1.B.ii). To calculate the PR^minor^ the two halves are the two sides across the minor axis (right and left in **Figure 1** **.B.iii**). Possible ranges vary between 0 (indicating for maximum symmetry) and 1 (indicating for maximum asymmetry).

### Calculation of binarization

The binarization of each division as symmetric versus asymmetric was achieved by counting only divisions that are polarized more along the major axis than along the minor axis; 
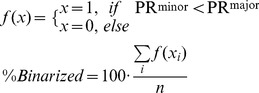
(Equation3)where f is result of specific binarized event, n is the number of divisions, and %*Binarized* is the percentage of division identified as polarized. These equations were used in Figure 5.C.

## Supporting Information

Figure S1 (TIF)

Figure S2 (TIF)

Figure S3 (TIF)

Figure S4 (TIF)

Figure S5 (TIF)

Text S1 (DOCX)

Data S1 (ZIP)

Data S2 (ZIP)

Data S3 (ZIP)

Data S4 (ZIP)
