# Supplementary figures and images for: Normalized Polarization Ratios for the Analysis of Cell Polarity
Source: PLoS One. 2014 Jun 25;9(6):e99885. doi: 10.1371/journal.pone.0099885 (PMC4070888; doi:10.1371/journal.pone.0099885)

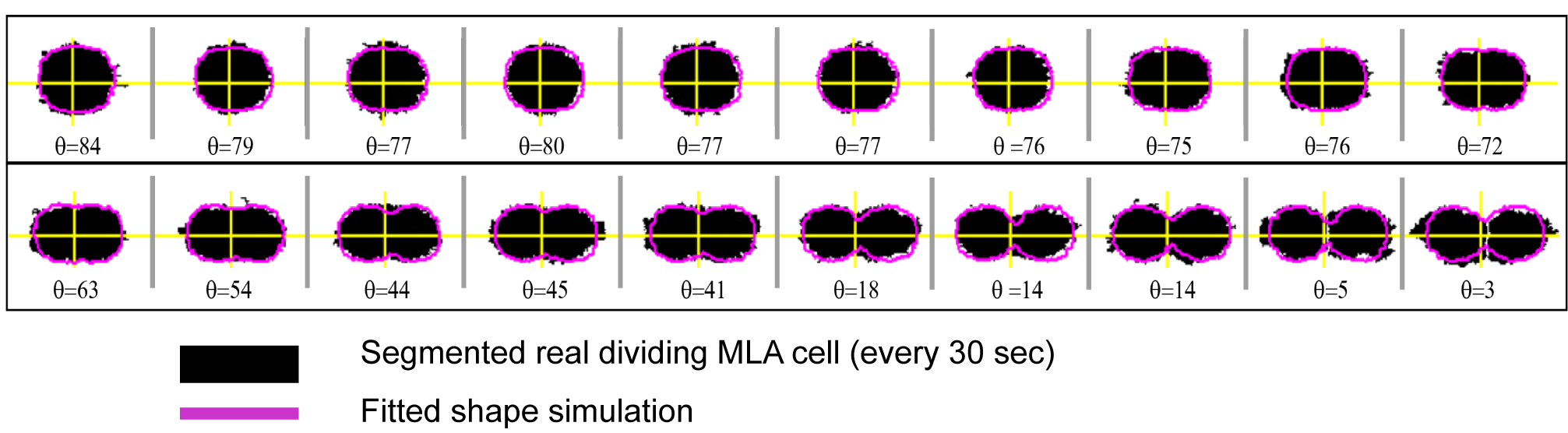

Supplement: Figure S1 — (TIF) [file pone.0099885.s001.tif]

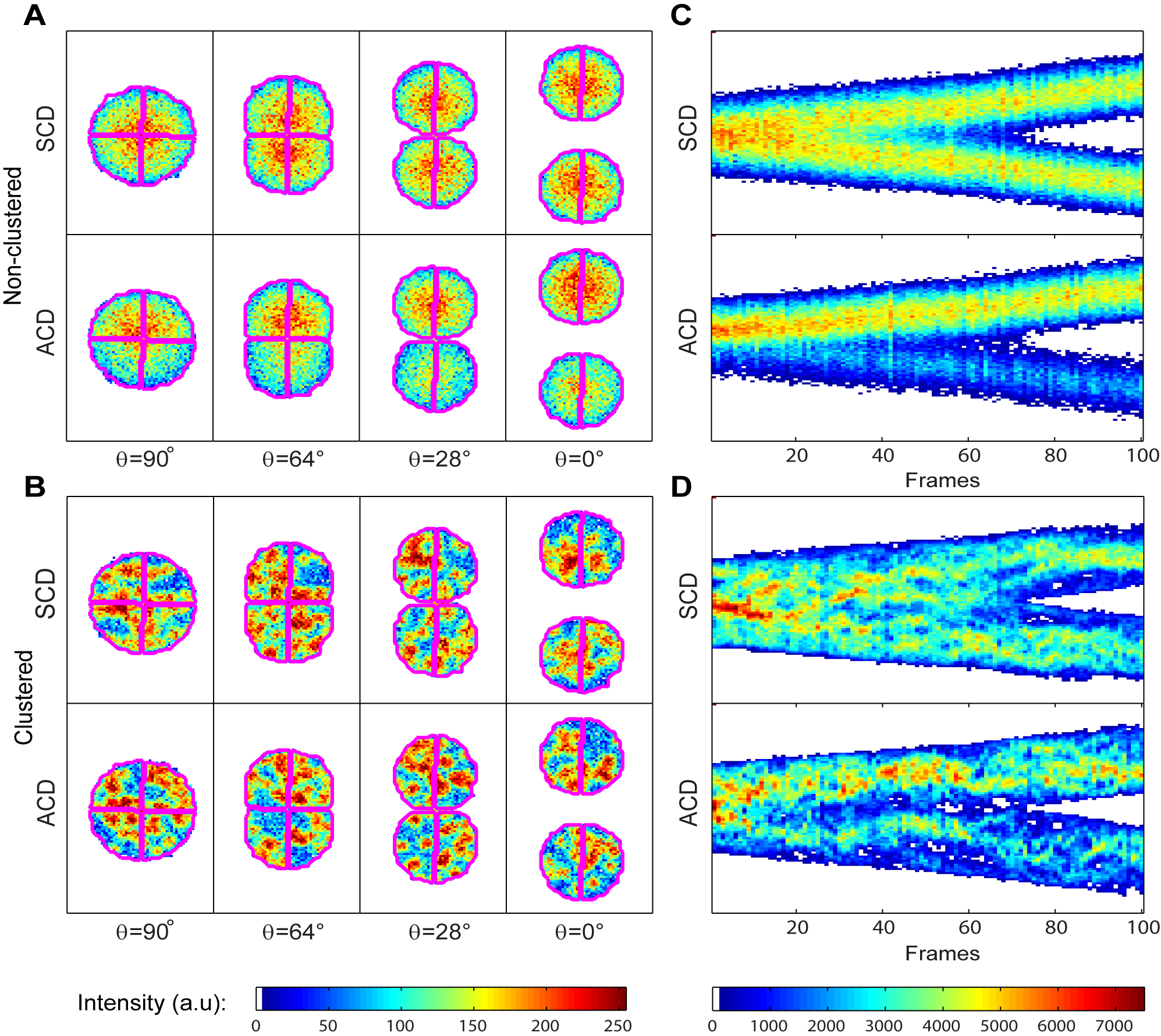

Supplement: Figure S2 — (TIF) [file pone.0099885.s002.tif]

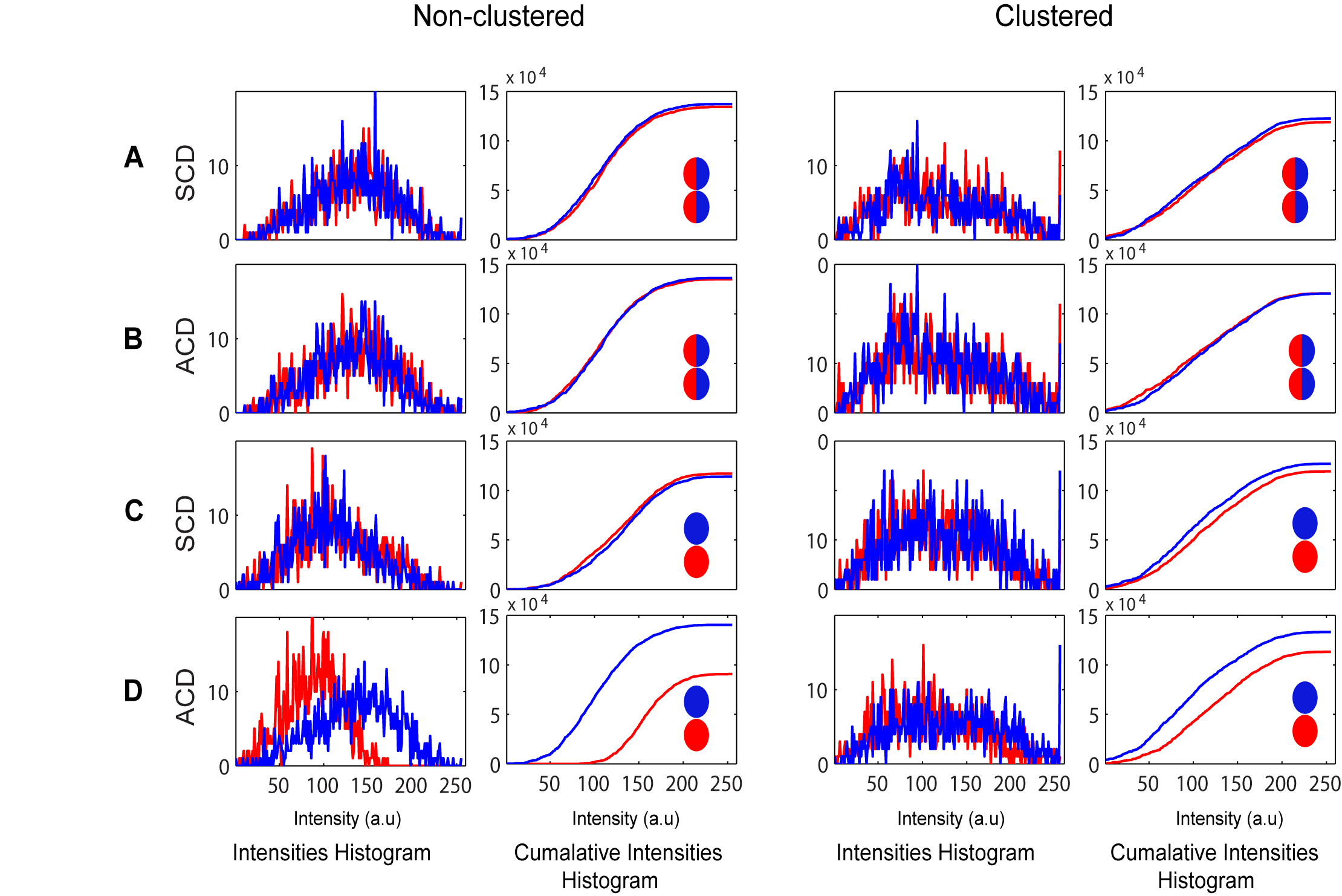

Supplement: Figure S3 — (TIF) [file pone.0099885.s003.tif]

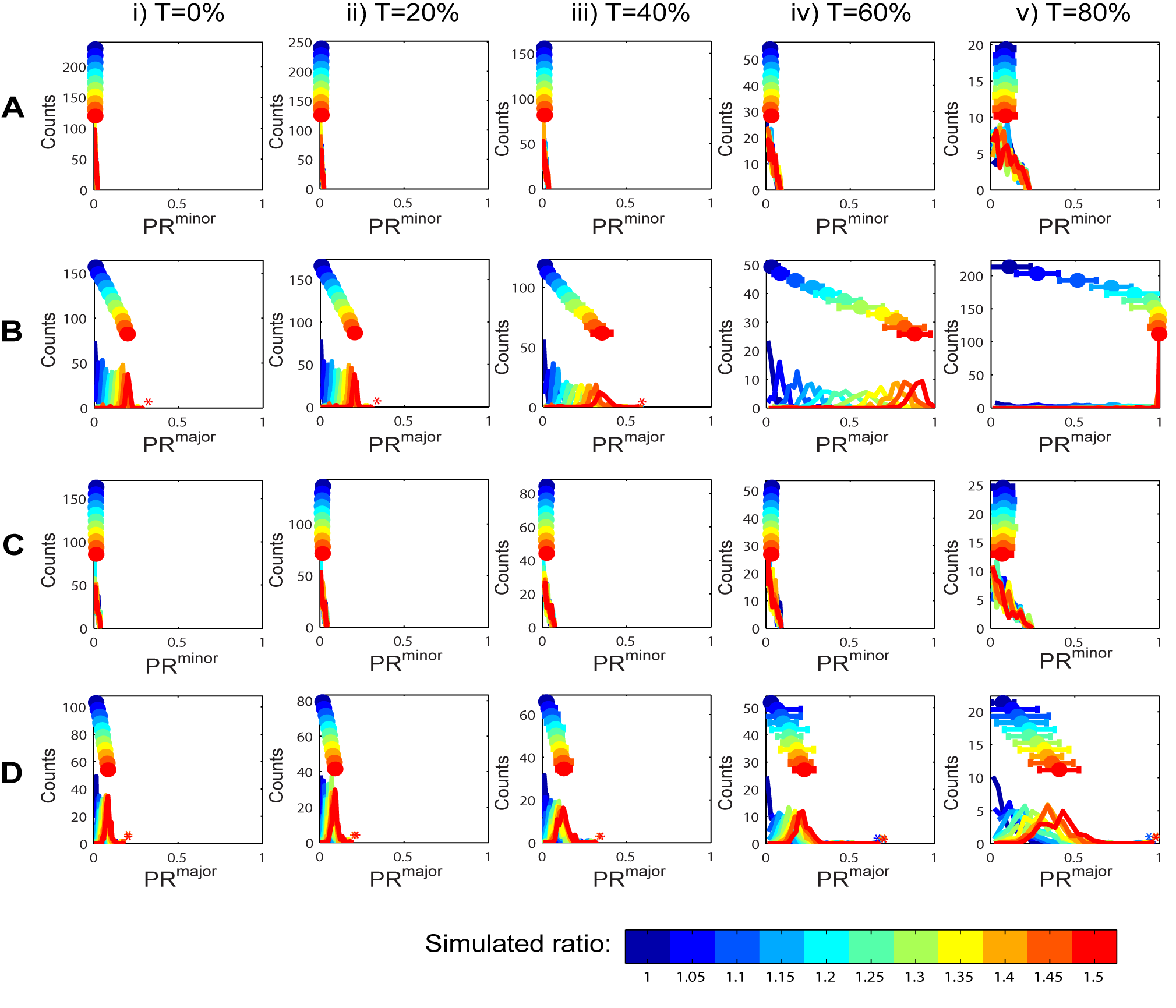

Supplement: Figure S4 — (TIF) [file pone.0099885.s004.tif]

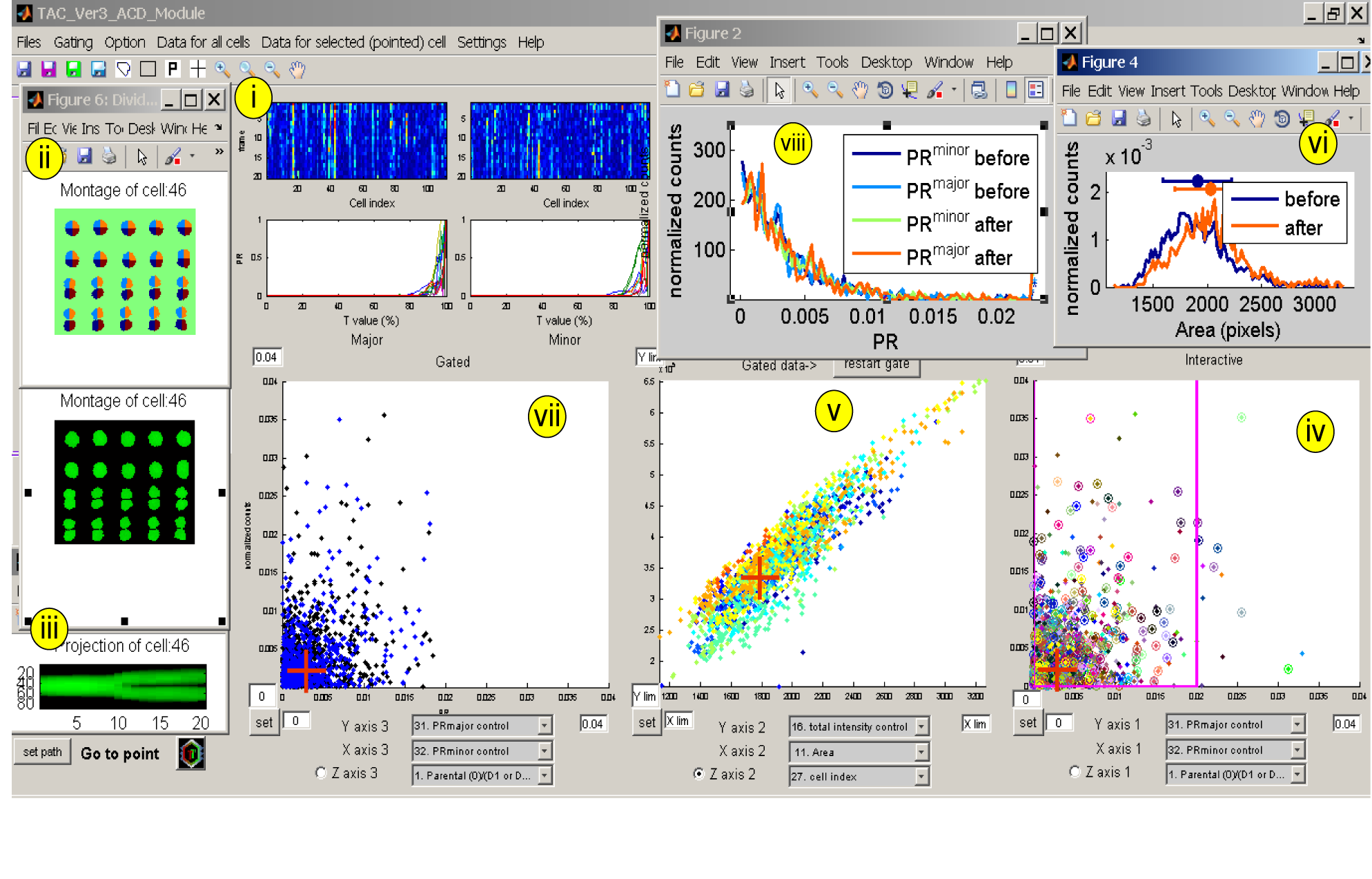

Supplement: Figure S5 — (TIF) [file pone.0099885.s005.tif]
