# Supplementary material for: Normalized Polarization Ratios for the Analysis of Cell Polarity
Source: PLoS One. 2014 Jun 25;9(6):e99885. doi: 10.1371/journal.pone.0099885 (PMC4070888; doi:10.1371/journal.pone.0099885)
Supplement: Text S1 — (DOCX) [file pone.0099885.s006.docx]

**Supporting Online Material**

**Binary image generation of cell division:**

Binary images of cell division were created by filling the perimeter of the simulated cells with ones pixels using the MATLAB function roipoly. To resemble to real cell divisions the perimeter of each division was modelled as two overlapped polygons allocated around the same centre. The left (Equation 1) and right side (Equation 2) polygons were created in x and y plane using the parametric equations:

Equation 1

$$\begin{matrix} x=r\cos(t_{1}+s_{1}+\frac{p_{1}}{100})+s_{1}+\frac{p_{2}}{100} \\ y=r\sin(t_{1}+s_{1}+\frac{p_{4}}{100})+s_{1}+\frac{p_{5}}{100} \\ 0\leq t_{1}\leq\pi\\ \pi\leq t_{2}\leq2\pi\end{matrix}$$

Equation 2

$$\begin{matrix} x=r\cos(t_{2}+s_{1}+\frac{p_{1}}{100})+s_{1}+\frac{p_{2}}{100} \\ y=(s_{2}+\frac{p_{3}}{100})r\sin(t_{2}+s_{1}+\frac{p_{4}}{100})+s_{1}+\frac{p_{5}}{100} \\ 0\leq t_{2}\leq\pi\\ \pi\leq t_{1}\leq2\pi\end{matrix}$$

where r is the equivalent radius of the polygons, s1=0.12 and s2= 1.1 are correction factors that were fitted into real data to give a slight stretch of the major axis along the x-axis (see supporting information Fig. S1). To generate various combinations of random shapes the model parameters included p1,p2,p3,p4,p5 random values from the standard uniform distribution on the open interval (0,1). The equivalent radius was estimated as function of the overlap between the two polygons:

Equation 3

$$r(\theta)=R\left(1+\frac{1}{2}\cos\theta\cdot(3-\cos^{2}\theta)\right)^{-1/3}$$

$$0\leq\theta\leq\frac{\pi}{2}$$

where R is the radius of the parental cell before the creation of the furrow, θ is the angle between x-axis and the intersection point between the two circles. In a real division, θ is function of the division stage. When θ is equal to zero the two polygons completely overlap to present the pre-divided parental cell, and as θ increases, the overlap is decreasing simulating higher development stage. All images were in size of 201 by 201 pixels, whereas the initial coordinates of the centre of each polygon were at (0,0). The shift of each cell from the centre is equal to half of the distance between the centroids of each daughter cell d, and was determined using the Law of cosines:

Equation 4

$$d=s_{3}\sqrt{2r^{2}(1-\cos(\pi-2\theta))}$$

$$0\leq\theta\leq\frac{\pi}{2}$$

where S3=1.1 is correction factors that fitted into real data in similar to s1 and s2.

**Supplementary Figure1. Comparison of simulated shape and an image of a dividing cell**. The magenta contour showing the goodness of the fit of the polygons equation compared with detected cell edges. Time points are 30 seconds between frames. θ was manually adjusted to give better fit. The R was 22 pixels.

**Supplementary Figure2. Simulations of cytokinesis.**

**(A, B)** Representative images of cells for clustered and non-clustered, symmetric and asymmetric as shown , in 4 different division stages. ACD was simulated by introducing different mean of fluorescence value (non-clustered) or number of clusters between the two daughter cells**. (C, D)** Intensity projections along the major axis of each cell over 100 frames. Visualization of such heat maps can display qualitative differences between the two daughter cells. **(A)** shows representative synthetic images of cells with non-clustered localization undergoing division at four different developmental stages: 90, 64, 28, 0, degrees. Cells are aligned along the major axis of each division. The ratio of total pixel intensity in the two daughters was 1:1 in the symmetric and 1:1.5 in the asymmetric cell division. The magenta coloured contours show how cells were subdivided and the border of the cells. Arbitrary units show projected sum intensity, and pixel intensity is colour coded according to the colour bars. The parental radius before division was 22 pixels. The average width of the clusters was 1.5 with standard deviation 0.02. For the aim of visualization, images were cropped to remove part of the image sides that do not include part of the cells. The increased intensity in the centre of the cells is due to the radial distribution function that was introduced to mimic volume effect. **(B)** shows representative synthetic images of a cell with clustered localization undergoing division presented as in (A). The numbers of clusters in the two daughters was 40 in the symmetric and 40 and 20 in the asymmetric cell division, which corresponds to ratios of 1:1 and 1:1.5 respectively. **(C)** shows a two- dimensional projection that gives qualitative information about progression of asymmetry/symmetry fluorescence partitioning of the same cell shown in **(A)**. Each column represents the total fluorescence intensity of individual cells for a simulated division. The projections are composed of 100 sequence frames that represent the progression of the cell division, whereas θ varies from 0 to 90 degrees in even increments. The representative images in **(A)** (90, 64, 28, 0, degrees) that corresponding to frames 1, 30, 70, 100 in respectively. **(D)** shows pixels with values lower than 140 (a.u.) were discarded to remove background and basal intensities from the projections. The MATLAB source-code of these simulations is provided as Supplementary Material 4).

**Supplementary Figure3. Intensities histograms corresponding to Figure 3.** Intensity histograms of 4 simulated divisions that corresponding to the 4 cases illustrated in Figure 4 A-D. Pixel intensities for the two daughter cells (D1 and D2) and for the two subsections of the division (L and R) were set in intensity histograms starting from the maximum pixel intensities (255) to the minimum (0). The blue and red lines represent the two opposite sides.

**Supplementary Figure4.** 100 cell division were simulated in increasing ratios from 1 to 1.5, giving 1100 divisions in total as represented in "jet" colors (blue to red). Data corresponds to Figure 7, non-clustered (**A** and **B**) and clustered (**C** and **D**). Data is showed counting histogram of PR^minor^ (**A** and **C**) and PR^major^ (**B** and **D**), under range of threshold from T=0%, to T= 80% in increments of 20%. Magenta line shows gating exclusion of 10% of data with high PR^minor^.

**Supplementary Figure5. Screen shot of TACTICS ACD Module**

Screenshot and analysis using the TACTICS ACD Module. Time-lapse fluorescence images are processed automatically using our high content and multi-dimensional data analysis TACTICS Toolbox (available in [TACTICS-Toolbox.com](file:///C:\Users\Olga\Desktop\TACTICS-Toolbox.com)). Briefly, TACTICS is a computational platform for high content analysis of time lapse images that specializes in immune cells [[22](#_ENREF_22),[38](#_ENREF_38),[39](#_ENREF_39)]. As a paradigm for the utilization of TACTICS, MLA expressing GFP (from the same data set shown in Figure 1) were imaged undergoing symmetric cell division. Time sequences were segmented, labelled and tracked over time using TACTICS Toolbox as described previously [[22](#_ENREF_22)], and PR ratios were measured using our approach. The steps for the analysis describe as the following: i) screening the PR as function of threshold values in incremental steps allowing to systematically determinate the appropriate level. ii) Interactive tools allowing the user to inspect specific data points or cells that can be removed from the final analysis using a gating approach. Representative images of an MLA cell expressing GFP undergoing symmetric division in different development stages: Cell is elongated, the creation of the cleavage furrow. Daughter cells dissociate from each others. iii) Time projections of fluorescence intensity for selected cell. iv) Plotting the PR^major^ vs. PR^minor^ allows removal of aberrant divisions with high PR^minor^. For the aim of visualization, to give better separation the axis limits were set in different values between [0 0.04]. Color labelling of data points shows cells at different time points. In the example above, the cut-off was chosen to be 0.02. v) In addition to PR values, other parameters can be visualized. In this example, the cell intensity is plotted as function of area, and is colour as function of the development stage. vi) Scatter plot show profile compare the occurrence of area before and after the disassociation. It can be seen that in later stages of division area is increased, as predicated. vii) Scatter plot and ;viii) histogram comparing the PR values of gated data points. Slight difference between early and later stages of division but not between PR^major^ and PR^minor^ . Since GFP is expected to give symmetric ratio, this difference is possibly duo to the lost of internal asymmetry in later stage of cytokines (the change in morphology prior to the cell migration), or resulted from of out-of-focus effect (i.e. one of the daughter cells is imaged out-of-focus). The subtle differences in polarization ratio that can be detected in regardless settings applied highlights the value of our approach for assessing multiple frames to improve the accuracy of imaging, as the decision what time point during the division accurately characterize the point of division.

**Supplementary Data 1. Data simulations of SCD.**

Contains:

1. data_no_clustered_SCD.mat : synthetic images of symmetric division for no clustered expression.
2. data_clustered_SCD.mat : synthetic images of symmetric division for clustered expression
3. data_BW_SCD.mat : sub-section of the two types of expression for the PR assessment

This data is shown in Figure 3.

The utilization of this data require to be unzipped the Supplementary Material 1.zip file (file size ~1Mb) and to run the files in 1-3 MATLAB.

**Supplementary Data 2. Data simulations of ACD and SCD.**

Contains:

1. Extracted data from 10 simulated cells, both asymmetric and symmetric, for non-clustered and clustered, from each section. In total 16 files in MATLAB format (.mat).
2. PR for simulations.xls is contains PR values calculated from 1.
3. Representative images of ACD and SCD, for non-clustered and clustered.

This data is shown in Figure 4.

The utilization of this data require to be unzipped the Supplementary Material 2.zip file (file size ~417Kb) and to run the files in 1-2 MATLAB, and 3 in EXCEL.

**Supplementary Data 3. Source-code for robust simulations.**

Supplementary Material 3.zip contains the MATLAB source-code for robust simulation used in Figure 5-7. The data simulation requires transfer of 500 Mb and is available upon request. To run the simulations:

1. Unzip the Supplementary Material 3 file (file size ~20Kb).
2. Lunch the file robust_simulation.m data_no_clustered_SCD.mat in MATLAB. The next function will be used in the following order:
   1. FIRST_PARENTAL_Radius.mat
   2. FIRST_PARENTAL_TOTAL_INTENSITY.mat
   3. timebar_TACWrapper.m
   4. gendist.m
   5. Perlin.m
   6. get_oval_cell.m
   7. SCD_SIM_SPLIT.m
   8. South_North_function.m
   9. Customgauss.m
   10. Spotmaker.m
   11. bad_data_function.m
3. The simulations take a few minutes on a standard personal laptop.

**Supplementary Data 4. Source-code for robust simulations.**

Supplementary Data 4 contains the MATLAB source-code for simulations of SCD and ACD and the time projections used in Figure S2. The data simulation requires transfer of 100 Mb and is available upon request. To run the simulations unzip the Supplementary Material 4.zip file (file size ~93Kb, and lunch the file robust_simulation.m data_no_clustered_SCD.mat in MATLAB. The next function will be used in the following order:

1. Lunch clustered.m or non_clustered.m, this will open the user interface.
2. In the user interface go from main to: >>Run and store>SCD>go and >>Run and store>ACD>go.
3. From main go to: >>Plot
4. From main go to: >>Extract position and plot as figure allowing to save the figure for further analysis.
